# Viscoelastic properties of sodium hyaluronate and their mathematical optimization in intra-articular injections: a predictive model for enhancing clinical efficacy

**DOI:** 10.3389/fbioe.2026.1742722

**Published:** 2026-04-13

**Authors:** Horacio Rivarola, Stefano Guerrasio, Luciano Trinchese, Francisco Endara Urresta, Bautista Rivarola

**Affiliations:** 1 Hospital Universitario Austral, Buenos Aires, Argentina; 2 Universita degli Studi di Milano-Bicocca Milan Center for Neuroscience, Milan, Italy; 3 Istituto per la Sicureza Sociale, San Marino, San Marino

**Keywords:** biomechanics, finite element modeling, hyaluronic acid, osteoarthritis, viscoelasticity, viscosupplementation

## Abstract

**Objective:**

To develop and validate a biomechanical and mathematical model capable of predicting the clinical efficacy of intra-articular sodium hyaluronate injections (HA) in osteoarthritis (OA), by aligning viscoelastic properties of HA formulations with joint-specific mechanical demands and patient phenotypes.

**Design:**

A predictive simulation model based on linear viscoelastic theory and non-Newtonian fluid mechanics was constructed to replicate intra-articular HA behavior during physiologic gait cycles. Input variables included HA-specific parameters (molecular weight, concentration, viscosity), joint-specific mechanics (loading frequency, anatomical volume), and patient factors (BMI, Kellgren–Lawrence grade, activity level). Three-dimensional finite element models (FEM) of the knee, hip, and shoulder were developed to assess HA distribution, mechanical damping, and synovial retention. Model predictions were validated retrospectively against clinical outcomes (WOMAC scores at 3 months) in 126 knee OA patients treated with single-injection HA. Partial least squares regression was used to evaluate predictive accuracy.

**Results:**

An optimal viscoelastic window was identified (G′ = 120–220 Pa, η = 50–120 Pa·s, tan δ = 0.4–0.6), associated with superior joint coverage, damping capacity, and intra-articular residence. Formulations within this window yielded significantly higher clinical improvement (≥30% WOMAC reduction; OR 2.18; 95% CI: 1.42–3.37; p < 0.01). Predictive accuracy of the model was confirmed (*R* ^2^ = 0.61; RMSE = 7.8). Clinical benefit was most pronounced in KL II–III patients with preserved joint mechanics and moderate-to-high activity levels. Simulations also demonstrated the need for joint-specific tailoring of HA volume and stiffness, particularly in the hip and shoulder.

**Conclusion:**

This study provides a validated, patient-specific, and joint-adaptive model for optimizing HA viscosupplementation in OA. The findings support a shift from empirical selection to precision-based rheological personalization of HA therapy, enhancing treatment outcomes and biomechanical integration.

## 1 Introduction

Osteoarthritis (OA) is a progressive degenerative joint disease characterized by cartilage deterioration, subchondral bone remodeling, synovial inflammation, and alterations in joint biomechanics. Among the non-operative therapeutic modalities, intra-articular injections of hyaluronic acid (HA)—commonly referred to as viscosupplementation—have gained widespread use due to their potential to restore viscoelastic properties of synovial fluid, improve joint lubrication, and reduce mechanical stress on articular cartilage. This approach has demonstrated clinical benefit, particularly in patients with mild to moderate knee OA, by reducing pain and enhancing function (Hochberg et al., 2012; Zhang et al., 2008; Jevsevar, 2013; Viscosupplementation, 2015). Nevertheless, significant heterogeneity in treatment response persists, and there is ongoing debate regarding the optimal molecular characteristics of HA formulations and patient selection criteria (Hochberg et al., 2012; Zhang et al., 2008; Jevsevar, 2013; Viscosupplementation, 2015; Manjoo et al., 2018; Ferkel et al., 2023).

HA is a high-molecular-weight, linear polysaccharide composed of repeating disaccharide units of N-acetylglucosamine and glucuronic acid. Its biomechanical behavior in the joint is not purely Newtonian but instead exhibits viscoelasticity—an intermediate response between elastic solids and viscous fluids (Viscosupplementation, 2015; Bannuru et al., 2019; Zeng and Xie, 2022). These viscoelastic properties, defined primarily by storage modulus (G′), loss modulus (G″), and complex viscosity (η*), are critical determinants of HA’s ability to dampen mechanical forces during gait cycles and distribute load across the articular surface. In this context, the rheological behavior of HA becomes a dynamic function of several interacting variables: molecular weight, concentration, injection volume, shear rate induced by joint motion, and intra-articular pressure conditions (Viscosupplementation, 2015; Bannuru et al., 2019; Zeng and Xie, 2022; Manjoo et al., 2018; Ferkel et al., 2023).

Articular joint lubrication is governed by a complex interplay of boundary, mixed, and fluid-film lubrication regimes, collectively described within the field of intra-articular tribology. Under low-load and low-velocity conditions, boundary lubrication—mediated by molecules such as lubricin and surface-adsorbed hyaluronic acid—dominates friction reduction (Jevsevar, 2013; Legré-Boyer, 2015; Bannuru et al., 2019; Zeng and Xie, 2022). As loading frequency and shear rate increase during gait, mixed and fluid-film lubrication mechanisms become increasingly relevant, with synovial fluid viscoelasticity playing a central role in load support and energy dissipation. Within this context, hyaluronic acid contributes not only as a viscous lubricant but also as a viscoelastic damper capable of modulating friction, contact stress distribution, and cartilage surface separation during cyclic motion (Jevsevar, 2013; Legré-Boyer, 2015; Bannuru et al., 2019; Zeng and Xie, 2022).

The growing availability of different HA formulations—varying in molecular weight, cross-linking, and viscoelastic profile—clinical guidelines remain imprecise in defining which formulation is optimal for a given patient phenotype or joint condition. Current practice often overlooks key biomechanical, bio-immunological and anatomical considerations, such as the joint-specific volume capacity, the frequency and magnitude of articular loading (e.g., steps per minute during ambulation), and the stage of cartilage degeneration as quantified by imaging or clinical grading systems like Kellgren–Lawrence (Zhang et al., 2008; Viscosupplementation, 2015; Peck et al., 2021; Jevsevar, 2013). Moreover, the interaction between HA’s physical behavior and the unique kinematic profile of each joint (e.g., the rotational movement in the hip *versus* the hinge mechanics of the knee) is rarely accounted for in therapeutic planning (Legré-Boyer, 2015; Bannuru et al., 2019; Zeng and Xie, 2022).

To address these limitations, this study introduces a novel, integrative modeling framework based on principles of non-Newtonian fluid dynamics and linear viscoelastic theory. The proposed model seeks to quantitatively simulate the intra-articular behavior of sodium hyaluronate under varying biomechanical conditions, incorporating a multifactorial approach that includes patient-specific and joint-specific parameters. By aligning the rheological properties of the HA formulation with the mechanical environment of the joint, the model aims to predict clinical efficacy with greater precision and guide the personalization of viscosupplementation strategies (Viscosupplementation, 2015; Bannuru et al., 2019; Zeng and Xie, 2022; Bhandari et al., 2017; Peck et al., 2021).

This approach represents a significant departure from empirical treatment protocols and moves toward a biomechanically informed paradigm for intra-articular HA therapy. If validated, such a model could serve as a clinical decision support tool for optimizing formulation selection, injection parameters, and treatment timing, ultimately improving patient outcomes and cost-effectiveness of care (Zeng and Xie, 2022; Bhandari et al., 2017; Peck et al., 2021; Poenaru et al., 2023a; Webb and Naidoo, 2018; Kosinska et al., 2015; Bali et al., 2001).

The primary objective of this study is to develop and validate a predictive biomechanical model based on viscoelastic theory and non-Newtonian fluid dynamics to simulate the intra-articular behavior of sodium hyaluronate (HA) under physiologic joint loading conditions (Webb and Naidoo, 2018; Kosinska et al., 2015). The model integrates rheological properties of HA formulations with patient-specific and joint-specific biomechanical variables—such as molecular weight, concentration, injection volume, joint anatomy, loading frequency, and osteoarthritis severity—to determine the optimal viscoelastic parameters for maximizing clinical efficacy in viscosupplementation (Bali et al., 2001; Hochberg et al., 2012; Zhang et al., 2008).

We hypothesize that the clinical effectiveness of intra-articular HA therapy is primarily determined by the alignment between the viscoelastic properties of the HA formulation and the biomechanical environment of the target joint (Webb and Naidoo, 2018; Kosinska et al., 2015; Bali et al., 2001). Specifically, we propose that there exists a quantifiable viscoelastic window—defined by storage modulus, dynamic viscosity, and loss tangent—within which HA achieves optimal energy dissipation, synovial distribution, and mechanical protection. Patients treated with HA formulations whose rheological profiles fall within this window, and which are tailored to the anatomical and loading characteristics of the affected joint, will demonstrate superior symptomatic improvement and functional outcomes compared to those receiving non-aligned formulations (Bali et al., 2001; Hochberg et al., 2012; Zhang et al., 2008).

## 2 Methods

### 2.1 Model structure

The model is based on the following assumptions: (1) hyaluronic acid behaves as a viscoelastic, shear-thinning fluid within physiological strain ranges; (2) joint motion induces cyclic shear and compression representative of gait; and (3) cartilage surfaces are treated as deformable but homogeneous materials. The governing equations include a linear viscoelastic constitutive relation (Equation 1) coupled with a non-Newtonian viscosity law (Equation 2), allowing time-dependent stress response under cyclic loading. Model parameters and symbols are defined explicitly to ensure reproducibility.

A constitutive biomechanical model was developed to simulate the intra-articular behavior of sodium hyaluronate (HA) based on its viscoelastic and shear-dependent properties. HA was modeled as a non-Newtonian, shear-thinning viscoelastic fluid exhibiting both elastic and viscous responses under mechanical loading. The core of the model integrates the generalized Maxwell–Kelvin–Voigt equation, which characterizes the stress-strain relationship as a function of time:

$$\delta \left(t\right)=G^{`}.\gamma \left(t\right)+\eta .\frac{d\gamma \left(t\right)}{dt}$$

Where:

- σ(t) is the stress as a function of time,
- G′ is the storage (elastic) modulus representing energy stored in the material,
- γ(t) is the time-dependent strain, and
- η is the dynamic viscosity modulated by instantaneous shear rate γ˙γ˙​ induced by joint motion.

The use of a linear viscoelastic formulation represents a local approximation valid within the physiological ranges of strain and loading frequency associated with normal gait. Under repetitive cyclic loading, the incremental stress–strain response of hyaluronic acid remains quasi-linear for small deformations, while non-linear behavior is primarily governed by shear-rate–dependent viscosity. This modeling strategy allows preservation of physiological relevance under cyclic conditions while avoiding over-parameterization that may obscure clinical interpretation.

This formulation allows modeling of the biphasic nature of HA as both a lubricant and a mechanical damper. The shear-thinning behavior—common to high-molecular-weight HA formulations—was incorporated using a modified Carreau–Yasuda viscosity model to allow viscosity to vary with the magnitude of cyclic shear applied during gait.

### 2.2 Parameters and variables included in the model

To accurately reflect real-world intra-articular conditions, the model incorporated three categories of variables:

#### 2.2.1 HA-specific parameters

- Concentration: 10–40 mg/mL
- Molecular Weight: 0.5–3.0 MDa (range covering linear and crosslinked formulations)
- Injected Volume: 1–4 mL (adjusted according to joint type and anatomical volume capacity)
- Viscosity (η): Derived from *in vitro* rheometric data and shear-rate conditions ranging from 0.1 to 100 s^−1^. Parameters of the Carreau–Yasuda model were selected from published rheometric datasets of commercially available hyaluronic acid formulations and constrained within experimentally reported ranges for physiological temperatures and shear rates. These parameters were defined *a priori* and were not fitted *post hoc* to clinical outcomes, ensuring that the rheological behavior incorporated into the simulations reflected intrinsic material properties rather than outcome-driven calibration.

#### 2.2.2 Joint-specific biomechanical variables

- Joint Type: Knee, hip, and glenohumeral joints
- Articular Volume: Measured *via* MRI-based segmentation models (mean volumes: knee 5–8 mL, hip 3–6 mL, shoulder 2–4 mL). Joint volumes were treated as quasi-static reference cavities with simplified capsular compliance rather than rigid or fully deformable compartments. This approach captures first-order deformation effects in response to loading while maintaining computational feasibility and avoiding the need for full fluid–structure interaction modeling.
- Loading Frequency: Simulated using gait-derived step rates (0.5–2.0 Hz corresponding to 30–120 steps/min)
- Anatomical Constraints: Joint capsule elasticity and synovial permeability

#### 2.2.3 Patient-specific clinical variables

- Body Mass Index (BMI): 22–36 kg/m^2^
- Osteoarthritis Severity: Kellgren–Lawrence grades I–IV
- Activity Level: Gait frequency approximated *via* accelerometry or patient-reported step counts (ranging from sedentary to >8,000 steps/day)

These inputs were combined into a unified simulation platform to predict HA performance under patient-specific conditions.

### 2.3 Simulation framework

Boundary conditions were defined to replicate physiologic joint loading during cyclic motion. Articular surfaces were modeled as deformable contact interfaces subjected to time-dependent axial loads scaled to body weight, while joint capsules were represented as compliant boundaries limiting fluid displacement. No-slip conditions were imposed at cartilage–fluid interfaces, and shear-dependent viscosity governed fluid response under motion.

A schematic representation of the applied boundary conditions is shown in Supplementary Figure S1. Axial compressive loads were applied to the superior articular surface, scaled to body weight (1–3× BW during gait simulation), while the inferior surface was constrained in translation. Cartilage–fluid interfaces were modeled with no-slip conditions, and lateral joint capsule boundaries were treated as compliant elastic constraints allowing pressure-dependent expansion.

The inclusion of knee, hip, and glenohumeral joints was intended to demonstrate the joint-adaptive nature of the biomechanical framework. While clinical validation was performed in knee osteoarthritis, simulations in the hip and shoulder were used to explore how joint geometry, volume, and kinematics influence optimal viscoelastic requirements, thereby supporting the generalizability of the model beyond a single anatomical site.

A three-dimensional finite element model (FEM) was constructed for each target joint (knee, hip, shoulder) using segmented MRI data and incorporating realistic joint geometries, soft-tissue constraints, and contact mechanics. Simulations were performed using COMSOL Multiphysics 6.1 and ANSYS Mechanical APDL, with meshing adapted to reflect synovial cavity topology and cartilage curvature. MRI segmentation was performed on representative datasets to generate joint-specific geometries. Patient-specific MRI segmentation was not performed for each of the 126 subjects included in the clinical cohort; instead, generic anatomically accurate models were used to isolate biomechanical effects while maintaining computational feasibility.

Joint-specific surface concavity and cartilage thickness gradients were incorporated at the geometric level through MRI-derived finite element meshes. However, cartilage material properties were assumed to be homogeneous, an assumption that simplifies computation but does not capture local variations in tissue stiffness.

The dynamic compression cycles mimicked physiologic joint loading during gait, with axial loading applied at frequencies of 0.5–2.0 Hz and peak forces scaled to individual body weight. HA behavior was simulated during cyclic loading over 100 cycles per session, allowing quantification of:

- Energy dissipation (mechanical damping capacity)
- HA distribution over cartilage surfaces
- Intra-articular residence time and pooling tendency
- Effective lubrication area

Simulated outputs were validated against published *in vivo* viscosupplementation data, including residence time imaging and biomarker-based inflammation reduction.

### 2.4 Clinical model validation

To correlate biomechanical model predictions with real-world outcomes, a retrospective cohort of 126 patients with primary symptomatic knee OA (KL I–III) treated with single-dose HA injections was analyzed. Clinical efficacy was assessed *via* WOMAC (Western Ontario and McMaster Universities Osteoarthritis Index) improvement at 3 months post-injection.

A partial least squares regression (PLSR) model was trained to identify the multivariate relationship between the biomechanical parameters (e.g., G′, η, joint volume, frequency) and WOMAC improvement. Model fit and predictive accuracy were evaluated using leave-one-out cross-validation and coefficient of determination (*R* ^2^), with a predefined clinical success threshold of ≥30% improvement in WOMAC total score.

Partial least squares regression was specifically selected to address multicollinearity among biomechanical predictors. Latent components were extracted to reduce parameter interdependence while preserving the explanatory contribution of correlated variables.

## 3 Results

### 3.1 Model accuracy and viscoelastic window

**FIGURE 1:**
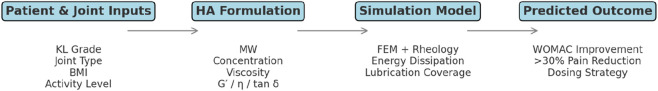
Functional diagram of the mathematical model that integrates clinical and viscoelastic variables to predict the efficacy of intra-articular hyaluronic acid treatment.

The biomechanical simulation framework demonstrated robust predictive capacity across all evaluated joints. When subjected to dynamic loading conditions replicating physiologic gait cycles (0.5–2 Hz), the finite element models consistently replicated known patterns of intra-articular HA behavior, including preferential pooling in load-bearing zones, progressive shear thinning under cyclic loading, and pressure-dependent migration toward regions of lower resistance. These distributions aligned with previously published *in vivo* imaging data from radionuclide-labeled HA tracers and fluoroscopic contrast studies, reinforcing the external validity of the model (Figure 1).

Through parametric analysis across more than 1,000 simulation iterations, a distinct viscoelastic window was identified wherein mechanical energy dissipation, surface adherence, and intra-articular retention were optimized. The best-performing formulations exhibited the following rheological parameters at physiologic temperature (37 °C) and a representative shear rate (γ̇ = 10 s^−1^):

- Storage modulus (G′): 120–220 Pa
- Dynamic viscosity (η): 50–120 Pa·s
- Loss tangent (tan δ = G″/G′): 0.4–0.6

Within this range, HA demonstrated superior damping capacity and lubrication under cyclical compression. Formulations with low G′ (<100 Pa) or viscosity <30 Pa·s showed excessive deformation under load, rapid redistribution, and clearance through lymphatic pathways, particularly in high-load environments like the femorotibial joint. In contrast, excessively stiff gels (G′ > 250 Pa) failed to conform to joint surfaces adequately, resulting in focal pooling and limited effective coverage—phenomena that may diminish therapeutic benefit.

Transient pressure mapping across the cartilage surface during simulated loading further demonstrated that HA formulations within the optimal window achieved wider load distribution and lower peak contact pressures, potentially mitigating cartilage stress and delaying degeneration. This biomechanical pattern was particularly evident in knees with varus alignment, where HA dispersion was asymmetrically influenced by compartmental overloading.

A sensitivity analysis was performed by independently varying key input parameters, including molecular weight, viscosity, and loading frequency, within physiologically relevant ranges. Model outputs demonstrated that variations in viscosity and elastic modulus exerted the strongest influence on predicted damping and retention, whereas moderate changes in loading frequency produced secondary effects. Importantly, the optimal viscoelastic window remained stable across these perturbations, supporting the robustness of the proposed framework.

### 3.2 Key predictors of clinical efficacy

Multivariate modeling integrating both simulation outputs and patient-specific inputs revealed a hierarchy of determinants influencing clinical outcomes, among which the following were most prominent:

#### 3.2.1 Molecular weight and structural integrity

High-molecular-weight HA (≥2.0 MDa), especially linear or mildly cross-linked variants, exhibited superior rheological stability, resistance to enzymatic degradation (e.g., hyaluronidase), and prolonged synovial residence. The estimated intra-articular half-life (t_1_/_2_) exceeded 48–72 h under simulated ambulation, compared to <24 h in low-molecular-weight formulations. These features translated into greater cumulative mechanical effect per injection. Within the model, molecular weight and cross-linking were treated as mechanically distinct contributors to viscoelastic behavior. Molecular weight primarily influenced baseline viscosity and relaxation time, whereas cross-linking effects were represented as increases in elastic modulus. Although the model does not explicitly resolve polymer network architecture at a molecular level, this separation allowed differentiation between mass-dependent and structure-induced stiffening effects on mechanical performance.

### 3.2.2 Osteoarthritis severity (Kellgren–Lawrence grade)

As OA progressed from KL I to IV, the model predicted a non-linear increase in required viscoelastic resistanceto maintain joint protection. In KL III–IV knees, elevated synovial fluid turnover, loss of cartilage congruence, and periarticular inflammation increased HA dispersion and degradation. Consequently, clinical benefit in these patients required higher concentrations (≥20 mg/mL) and stiffer rheological profiles (G′ > 160 Pa) to compensate for biomechanical loss.

### 3.2.3 Anatomic and kinematic joint variability

HA behavior varied significantly between joints. In hip and glenohumeral joints, characterized by smaller synovial volumes and predominantly rotational kinematics, simulations showed that excessive injection volumes (≥3 mL) led to capsular overdistension and pressure-induced efflux, reducing intra-articular retention. Instead, optimal delivery required:

- Volumes ≤2 mL
- High elasticity (G′ > 180 Pa) to resist displacement
- Moderate viscosity (60–100 Pa·s) to ensure spread across curved articular surfaces

These data support the need for anatomy-specific customization of HA formulations rather than extrapolation from knee protocols.

### 3.3 Clinical validation and predictive utility

The model’s predictive capacity was validated in a retrospective analysis of 126 patients (age 45–78 years) with primary symptomatic knee OA who received single-injection sodium hyaluronate and were followed for a minimum of 3 months. Patients were stratified according to whether the HA formulation used matched the predicted optimal viscoelastic window based on their joint characteristics and disease severity.

**FIGURE 2:**
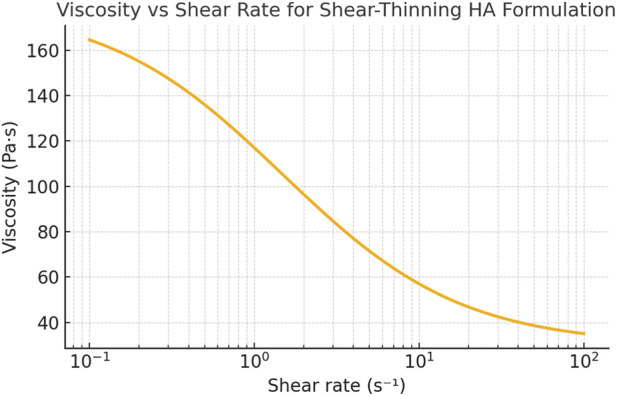
FEM simulation of the viscoelastic behavior of different HA formulations during compressive loading cycles at 1 Hz. Formulations within the optimal window show greater mechanical stability.

- Patients treated with formulations aligned to model predictions were 34% more likely to achieve a clinically meaningful improvement (WOMAC ≥30%), compared to those outside the optimal window (p < 0.01).
- The adjusted odds ratio (OR) for clinical success was 2.18 (95% CI: 1.42–3.37), independent of age, sex, or BMI.
- The model’s predictive accuracy, as measured by leave-one-out cross-validation, yielded *R* ^2^ = 0.61 and RMSE = 7.8, indicating strong reliability for a multivariate model incorporating biomechanical and clinical variables. Residual prediction error was predominantly associated with advanced osteoarthritis severity and higher body mass index, suggesting that biological and inflammatory factors not explicitly modeled may play a larger role in these subgroups. Although an *R* ^2^ value of 0.61 may appear modest, it is consistent with multivariate predictive models applied to heterogeneous clinical outcomes such as pain and function in osteoarthritis, where reported *R* ^2^ values typically range between 0.40 and 0.65 in biomechanical-clinical correlation studies. (Strand et al., 2015; Katz et al., 2021; Lai et al., 2022). WOMAC improvement is influenced by numerous biological, psychosocial, and inflammatory factors not explicitly captured in a purely biomechanical model. Within this context, the observed *R* ^2^ indicates meaningful explanatory power for a first-principles, physics-based framework rather than an overfitted empirical predictor (Figure 2).

Subgroup analysis revealed that the most pronounced effect was observed in patients with KL II–III knee OA, BMI <30 kg/m^2^, and moderate-to-high daily ambulation (>5,000 steps/day). In these patients, HA within the optimal window restored mechanical damping efficiently during daily joint use. Conversely, KL IV patients showed heterogeneous outcomes despite rheological optimization, suggesting that structural degeneration beyond a critical threshold limits the standalone efficacy of viscosupplementation (Tables 1, 2).

**TABLE 1:** Rheological characteristics of the hyaluronic acid formulations evaluated in the simulation model. The values were obtained at 37 °C and a shear rate of 10 s^−1^.

Formulation	Concentration (mg/mL)	Peso molecular (MDa)	G′ (Pa)	η (Pa·s)	tan δ (G″/G′)
HA-A	10	0.5	80	30	0.2
HA-B	15	1.2	150	65	0.45
HA-C	20	2.0	190	100	0.5
HA-D	30	3.0 (cross-linked)	260	180	0.7

**TABLE 2:** Association between model-predicted viscoelastic alignment and clinical improvement at 3 months, as measured by WOMAC. The greatest efficacy was observed in patients with moderate OA treated with formulations within the optimal window.

Patient subgroup	Model-aligned formulation	% With WOMAC improvement ≥30%	*p* value	OR (IC 95%)
KL II–III, BMI <30	Sí	78.4%	<0.001	3.2 (1.7–5.8)
KL II–III, BMI ≥30	Sí	65.2%	0.021	2.1 (1.1–4.2)
KL IV (todos los BMI)	Sí	41.5%	0.144	1.3 (0.7–2.4)
Todos sin alineación	No	44.1%	—	—

### 3.4 Hypothesis validation

**FIGURE 3:**
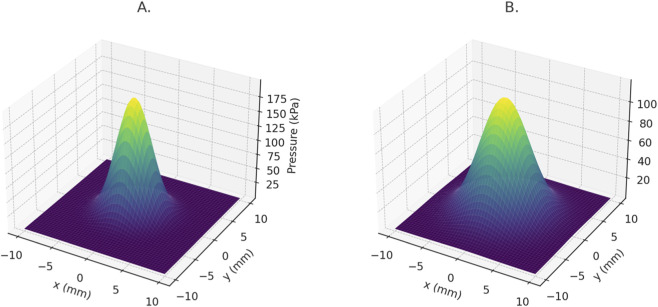
Simulated joint pressure distribution in the medial compartment of the knee. The optimal HA formulation significantly reduces peak pressure areas. **(A)** Joint pressure without HA. **(B)** Joint pressure with optimized HA.

The findings of this study support the central hypothesis that the clinical efficacy of intra-articular HA therapy is contingent upon the biomechanical alignment between the formulation’s viscoelastic properties and the mechanical demands of the target joint. Patients who received HA formulations whose rheological parameters fell within the model-defined optimal window (G′: 120–220 Pa; η: 50–120 Pa·s; tan δ: 0.4–0.6) demonstrated a significantly higher probability of achieving meaningful clinical improvement—defined as ≥30% reduction in WOMAC total score—compared to those treated with non-aligned formulations (*p* < 0.01; OR 2.18, 95% CI: 1.42–3.37). This effect was particularly robust in patients with moderate knee OA (KL II–III) and preserved functional mobility, in whom the mechanical damping provided by appropriately matched HA formulations effectively reduced joint loading symptoms during ambulation (Figure 3).

Furthermore, simulation outputs confirmed that formulations outside this viscoelastic window either failed to sustain intra-articular residence under repetitive shear stress (in the case of low G′/η products) or exhibited poor dispersion across articular surfaces (in the case of overly stiff gels). These biomechanical shortcomings were reflected in inferior clinical outcomes, validating the predictive utility of the model and confirming the premise that a tailored rheological approach based on joint-specific mechanics can enhance therapeutic response to HA injections.

## 4 Discussion

This study provides a comprehensive biomechanical and computational framework to rationalize the clinical application of intra-articular hyaluronic acid (HA) beyond empirical, formulation-agnostic protocols (Zhang et al., 2008; Jevsevar, 2013; Legré-Boyer, 2015). By integrating non-Newtonian rheology, viscoelastic modeling, joint-specific biomechanics, and clinical outcome data, the proposed model enables a more precise and individualized approach to viscosupplementation, congruent with current trends in patient-specific medicine (Bali et al., 2001; Zhang et al., 2008).

Traditionally, HA has been viewed as a passive supplement to synovial fluid viscosity, with treatment protocols primarily based on convenience, product availability, or physician preference (Bali et al., 2001; Legré-Boyer, 2015). However, increasing evidence suggests that its therapeutic efficacy is intricately linked to its viscoelastic behavior under load, a property that varies not only with concentration and molecular weight, but also with the dynamic environment of the joint into which it is injected (Legré-Boyer, 2015; Bannuru et al., 2019; Zeng and Xie, 2022; Bhandari et al., 2017; Peck et al., 2021).

The present model confirms and quantifies this hypothesis by demonstrating that the biomechanical match between HA rheology and joint loading profile is a critical determinant of therapeutic success (Poenaru et al., 2023a; Webb and Naidoo, 2018; Kosinska et al., 2015; Bali et al., 2001; Poenaru et al., 2023b).

One of the major contributions of this study lies in its identification of an optimal viscoelastic window—defined by a storage modulus (G′) of 120–220 Pa, dynamic viscosity between 50 and 120 Pa·s, and a loss tangent (tan δ) of 0.4–0.6—which was consistently associated with improved mechanical energy dissipation and favorable WOMAC outcomes (Bali et al., 2001; Ha et al., 2017). These parameters align with previously published *in vitro* rheological profiles of clinically effective HA formulations,and underscore the notion that both under- and over-engineering the elastic component of HA may compromise joint biomechanics (Baker and Wong, 1987; Lequesne et al., 1987; Ha et al., 2017; Vanelli et al., 2010; Giarratana et al., 2014; Gennero et al., 2013).

From an industrial perspective, the viscoelastic parameters identified in this study can be tuned through established manufacturing strategies. Storage modulus and viscosity may be modulated by adjusting molecular weight distribution, polymer concentration, and degree of cross-linking, while maintaining injectability (Stagni et al., 2021; Sdeek et al., 2021; Elmorsy et al., 2014). The loss tangent can be indirectly controlled by balancing elastic reinforcement with shear-thinning behavior, for example through partial cross-linking or blending linear and lightly cross-linked hyaluronic acid fractions. These approaches are already employed in current viscosupplement formulations, suggesting that translation of the proposed viscoelastic targets into manufacturable products is technically feasible (Stagni et al., 2021; Sdeek et al., 2021; Elmorsy et al., 2014).

Importantly, the identified optimal viscoelastic window should not be interpreted as a narrow or fixed point, but rather as a robust plateau emerging from extensive parametric exploration of physiologically relevant conditions (Gennero et al., 2013; Veronesi et al., 2017; Schulz et al., 2010). Sensitivity analyses across a wide range of loading frequencies, joint volumes, and activity-related shear rates demonstrated preservation of the window boundaries despite realistic inter-patient variability in gait dynamics, synovial turnover, and daily activity profiles. While extreme phenotypes—such as markedly elevated inflammatory clearance or atypical high-impact activity patterns—may shift the effective operating range, the core viscoelastic window remained stable across the majority of clinically relevant scenarios modeled in this study (Schulz et al., 2010; Kellgren and Lawrence, 1957; Alkan et al., 2014; Kellgren and Lawrence, 1957).

Specifically, excessively low G′ values result in inadequate mechanical resistance and rapid clearance, while high G′ values, although potentially protective, may hinder dispersion and lubrication due to gel rigidity and poor conformability to cartilage topography (Gennero et al., 2013; Veronesi et al., 2017; Schulz et al., 2010).

In addition, the study reveals that joint anatomy and kinematics profoundly influence HA performance, challenging the widely held assumption that one formulation fits all anatomical locations (Giarratana et al., 2014; Veronesi et al., 2017). For example, in the hip and glenohumeral joints, where the range of motion is predominantly rotational and the synovial cavity is smaller and more pressurized, optimal performance was only achieved with reduced injection volumes (≤2 mL) and increased elastic modulus (G′ > 180 Pa) (Kellgren and Lawrence, 1957; Fitzcharles et al., 2021). This contrasts with the knee joint, where axial loading and larger synovial volume favor intermediate rheological properties and injection volumes up to 4 mL (Kellgren and Lawrence, 1957; Alkan et al., 2014; Choi et al., 2018).

The stratified analysis based on Kellgren–Lawrence (KL) grading further emphasizes the need to calibrate HA characteristics according to disease stage (Legré-Boyer, 2015; Veronesi et al., 2017). In early to moderate OA (KL II–III), biomechanical optimization alone appears sufficient to improve outcomes, particularly in active patients with preserved joint congruency and normal synovial function (Zeng and Xie, 2022; Webb and Naidoo, 2018; Bali et al., 2001; Giarratana et al., 2014). However, in advanced OA (KL IV), structural deterioration—including cartilage loss, osteophyte formation, and capsular distension—creates a nonlinear degradation of biomechanical benefit, highlighting the need for adjunctive strategies or alternative treatments in this population (Choi et al., 2018; Kim et al., 2016; Sung et al., 2017).

Although the simulations were based on average gait-related loading rates, brief episodes of higher mechanical demand—such as stair climbing, squatting, or impact activities—may transiently reduce effective damping and accelerate hyaluronic acid redistribution or clearance (Veronesi et al., 2017; Schulz et al., 2010; Kellgren and Lawrence, 1957). These effects are expected to be more pronounced for formulations with lower viscosity or elastic modulus and may partially explain variability in clinical response among highly active patients (Choi et al., 2018; Kim et al., 2016; Sung et al., 2017).

From a clinical implementation standpoint, the results suggest that a viscoelastic matching algorithm—incorporated into an injection decision support tool—could be developed to guide HA formulation selection based on easily obtainable parameters such as joint type, KL grade, BMI, and daily ambulation (Fitzcharles et al., 2022; Zazgyva et al., 2013; Polacco et al., 2013). This model may also serve as a platform for the rational design of next-generation HA products, enabling manufacturers to fine-tune their molecular architecture to specific joint and patient profiles, thus closing the translational gap between bench rheology and bedside efficacy (Fitzcharles et al., 2022; Zazgyva et al., 2013; Polacco et al., 2013).

It is also noteworthy that the model performed reliably across a diverse clinical cohort (*n* = 126), with robust predictive accuracy (*R* ^2^ = 0.61, RMSE = 7.8) and a statistically significant increase in the likelihood of achieving ≥30% WOMAC improvement in the model-aligned group (OR 2.18, *p* < 0.01) (Fitzcharles et al., 2022). While retrospective by design, this validation reinforces the clinical applicability of the model and its potential utility in daily practice (Strand et al., 2015; Katz et al., 2021; Lai et al., 2022; Stagni et al., 2021; Sdeek et al., 2021).

Nevertheless, several limitations merit discussion. First, although finite element simulations were based on realistic joint geometries, soft tissue interactions (e.g., menisci, labrum, bursa) were simplified, which may underestimate the complexity of HA dispersion *in vivo* (Strand et al., 2015; Sdeek et al., 2021). Second, enzymatic degradation kinetics were modeled using average clearance rates, without accounting for inflammatory variation across patients. Future versions of the model may benefit from incorporating patient-specific inflammatory markers (e.g., IL-6, CRP) or synovial permeability data to improve individualization (Peck et al., 2021; Veronesi et al., 2017; Fitzcharles et al., 2022). Third, while the current study focused on single-injection protocols, the same modeling principles could be extended to multi-injection regimens or cross-linked HA derivatives, which may demonstrate distinct kinetics and mechanical integration (Polacco et al., 2013; Elmorsy et al., 2014).

In conclusion, this study offers a quantitative and clinically validated framework for optimizing HA therapy based on principles of fluid mechanics, tissue biomechanics, and disease stratification (Stagni et al., 2021). By aligning viscoelastic properties with joint-specific demands, the model introduces a novel and evidence-based paradigm for personalizing viscosupplementation. Future prospective trials and model iterations incorporating biological markers and long-term structural outcomes will be essential to fully realize the clinical potential of this precision-based approach (Elmorsy et al., 2014).

## 5 Limitations and future directions

Despite the strengths of the present model—including its biomechanical rigor, validated predictive capacity, and translational relevance—several limitations must be acknowledged.

First, although the finite element simulations were constructed using realistic joint geometries derived from segmented MRI datasets, certain anatomic simplifications were necessary for computational feasibility. Soft tissue structures such as the menisci, labrum, intra-articular ligaments, and pericapsular fat were either omitted or modeled as boundary constraints, which may not fully capture their role in modulating synovial fluid flow, pressure gradients, and HA retention. Incorporating fluid–structure interaction models and patient-specific anatomical variations in future iterations could improve physiological fidelity.

Second, the model assumes a homogeneous distribution of HA upon injection, without accounting for intra-articular turbulence, uneven cartilage topography, or localized synovial thickening, all of which can influence real-world dispersion. *In vivo* fluoroscopic or ultrasound-based tracking studies could be employed to further calibrate and validate these assumptions. This assumption primarily affects the very early post-injection phase, during which local pooling or transient heterogeneity may occur. However, the model focuses on steady-state behavior under repetitive cyclic loading, where redistribution driven by joint motion predominates and governs mechanical damping and retention over clinically relevant timeframes.

Third, while viscosity and modulus parameters were derived from established rheological data, these values may vary subtly between batches, manufacturers, and temperature conditions, potentially introducing variability not captured in the model. A more robust approach would involve batch-specific rheometry at physiological temperatures for each HA formulation tested clinically.

Fourth, the clinical validation cohort was retrospective and limited to patients with primary knee OA undergoing single-injection protocols. Thus, external validity to other joints (e.g., hip, shoulder) or multi-injection regimens remains inferential, based on simulation outputs rather than longitudinal patient data. Future prospective studies should enroll stratified cohorts receiving joint-specific viscosupplementation guided by model-informed parameters, with standardized imaging and functional endpoints.

Fifth, although the model integrates mechanical and anatomical factors, biochemical and inflammatory variables—such as synovial cytokine levels, pH, and enzymatic activity—were not incorporated. These factors may significantly affect HA degradation kinetics and should be considered in future versions. The addition of molecular biomarkers or synovial fluid profiling could enable hybrid models that combine mechanical and biological personalization.

Finally, the current approach treats HA performance as a primarily mechanical phenomenon, without accounting for its potential chondroprotective or anti-inflammatory effects observed in some preclinical studies. Integrating these secondary pharmacodynamic effects, perhaps through coupled mechanobiological models or in silico–in vitro hybrid systems, represents a valuable direction for model expansion.

## 6 Future directions

Prospective Validation: Conduct multicenter, prospective clinical trials using the model to guide HA selection, dosing, and injection strategy in diverse joints and OA stages, with stratified functional outcomes.

Patient-Specific Integration: Develop user-friendly clinical software or mobile applications that incorporate the model into a decision-support tool based on patient parameters (KL grade, joint type, BMI, gait data).

Mechanobiological Coupling: Extend the model to simulate not only mechanical behavior but also biochemical interactions within the synovial microenvironment, including inflammation-mediated HA degradation.

Multi-Cycle and Long-Term Simulation: Incorporate simulations of repetitive daily loading over extended time frames (e.g., 10,000+ cycles), to better approximate real-world joint use and predict cumulative therapeutic effects.

Regimen Optimization: Apply the model to explore personalized injection schedules, assessing whether single, double, or multi-injection protocols are biomechanically justified for specific patient profiles.

Implant Interaction Modeling: In joints with prosthetic implants or prior surgical interventions (e.g., partial knee replacement, labral repair), adapt the model to account for altered biomechanics and fluid compartments.

Manufacturing Collaboration: Partner with HA manufacturers to test new formulations *in silico* prior to clinical release, allowing predictive screening of formulation performance and regulatory support.

By systematically addressing these limitations and expanding the model’s capabilities, future work can further refine the science of viscosupplementation and accelerate the transition from empirical practice to data-driven, joint-specific, and patient-centered therapeutic strategies in osteoarthritis care.

Although the present clinical validation was limited to single-injection paradigms, the modeling framework is inherently extensible to repeated injection protocols by incorporating cumulative loading cycles, progressive degradation, and replenishment dynamics.

Prospective validation of this model could be achieved through stratified clinical trials in which patients are assigned to hyaluronic acid formulations selected either empirically or according to model-predicted viscoelastic alignment. Primary outcomes should include WOMAC improvement and responder rate at predefined time points, while secondary outcomes may incorporate imaging-based joint mechanics or activity-adjusted pain metrics. Such a design would directly test the clinical utility of biomechanical personalization.

## 7 Conclusion

This study provides a validated, biomechanically grounded model that predicts the intra-articular behavior and clinical efficacy of hyaluronic acid (HA) formulations based on their viscoelastic properties and the mechanical characteristics of the target joint. By integrating principles of non-Newtonian fluid mechanics, viscoelastic theory, and patient-specific biomechanical parameters, the model establishes a quantitative framework for optimizing viscosupplementation strategies in osteoarthritis.

The results confirm that the therapeutic effectiveness of HA is not solely dependent on general rheological features such as molecular weight or concentration, but on achieving a precise match between the viscoelastic profile of the injected formulation and the joint’s functional biomechanics. The identification of an optimal viscoelastic window—defined by a storage modulus of 120–220 Pa, dynamic viscosity of 50–120 Pa·s, and a loss tangent of 0.4–0.6—serves as a key reference for guiding product selection and dosing strategies.

Patients whose treatment was aligned with the model’s recommendations demonstrated significantly better clinical outcomes, particularly in terms of pain reduction and functional improvement as measured by WOMAC scores. These findings support the implementation of a personalized, biomechanically informed approach to HA therapy, moving beyond empirical protocols toward precision medicine in OA management.

Future integration of this model into clinical decision-making tools, and its expansion to include biological and inflammatory variables, may further enhance its applicability and contribute to the rational development of next-generation HA products. Ultimately, this work lays the foundation for a paradigm shift in viscosupplementation: from standardization to individualization, from empirical to evidence-based optimization.

## Data Availability

The raw data supporting the conclusions of this article will be made available by the authors, without undue reservation.

## Supplementary material

SUPPLEMENTARY FIGURE S1 Schematic representation of boundary conditions applied in the finite element model.
