# Supplementary material for: Viscoelastic properties of sodium hyaluronate and their mathematical optimization in intra-articular injections: a predictive model for enhancing clinical efficacy
Source: Front Bioeng Biotechnol. 2026 Apr 13;14:1742722. doi: 10.3389/fbioe.2026.1742722 (PMC13111471; doi:10.3389/fbioe.2026.1742722)
Supplement: Supplementary file 1 [file Supplementaryfile1.docx]

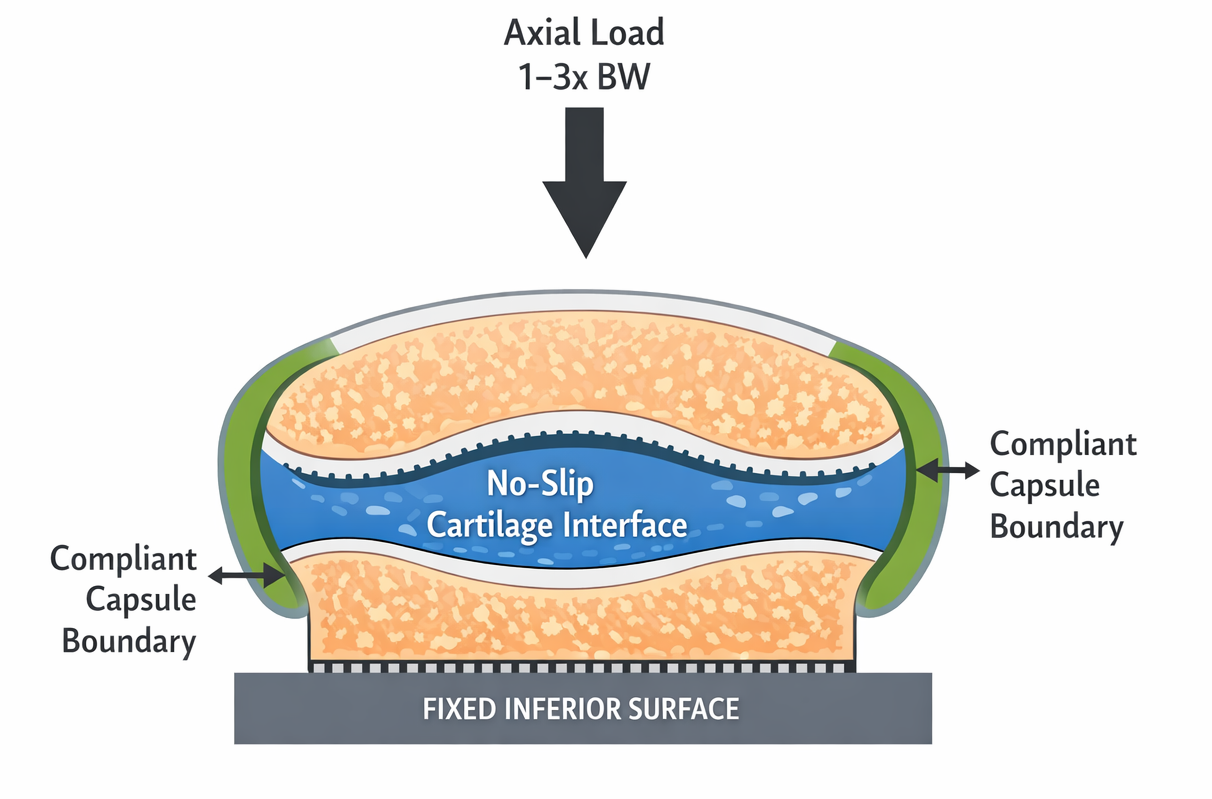


Figure S1. Schematic representation of boundary conditions applied in the finite element model.

The inclusion of knee, hip, and glenohumeral joints was intended to demonstrate the joint-adaptive nature of the biomechanical framework. While clinical validation was performed in knee osteoarthritis, simulations in the hip and shoulder were used to explore how joint geometry, volume, and kinematics influence optimal viscoelastic requirements, thereby supporting the generalizability of the model beyond a single anatomical site.
